# Self-Supervised Text-Driven Point Cloud Upsampling via Semantic Text Guidance

**DOI:** 10.3390/jimaging12050204

**Published:** 2026-05-11

**Authors:** Zhiyong Zhang, Meiling Qiu, Shuo Chen, Ruyu Liu, Jianhua Zhang, Shengyong Chen

**Affiliations:** 1 School of Computer Science and Engineering, Tianjin University of Technology, Tianjin 300000, China; 2 School of Information Science and Technology, Hangzhou Normal University, Hangzhou 310000, China; 3 Department of Technology, Management and Economics, Technical University of Denmark, 2800 Lyngby, Denmark

**Keywords:** biomimetic perception, point cloud upsampling, language-guided, vision–language model

## Abstract

Point cloud upsampling is a fundamental task in 3D vision, yet most existing methods adopt a global and uniform strategy, which is computationally inefficient and fails to address the need for region-specific refinement. To address this challenge, we propose PartSPUNet, a novel self-supervised, text-driven point cloud upsampling framework designed to enhance robotic perception through task-oriented local refinement. Inspired by the human cognitive process where high-level language instructions guide visual attention to specific regions of interest, our method allows an operator to use intuitive natural language prompts to direct the upsampling process. Specifically, PartSPUNet leverages a pretrained vision–language model to zero-shot localize the user-specified semantic part within a sparse point cloud. It then performs geometry-aware densification exclusively on this target region, recovering rich geometric details while preserving the global structure. Experimental results demonstrate that our approach significantly outperforms existing methods in reconstructing specified areas, offering a powerful and intuitive tool for enhancing the 3D perception pipeline in intelligent robotic systems.

## 1. Introduction

As a fundamental medium for representing three-dimensional geometry, point clouds see extensive use in fields ranging from object modeling [1] and virtual reality [2] to medical simulation [3,4]. Their utility derives from key characteristics, including an implicit topological structure, ease of acquisition, and a direct correspondence to object surface geometry. In practice, the quality of captured data, particularly for human subjects, is often limited. Factors such as sensor resolution constraints, lighting interference, and motion blur typically result in point clouds that are notably sparse and unevenly distributed. To address this, point cloud upsampling methods [5,6] have been developed. Such methods aim to reconstruct high-fidelity outputs from sparse, low-quality inputs, generating results that are dense, uniformly distributed, rich in surface detail, and topologically plausible. This process significantly enhances the visual quality and geometric completeness of the data, thereby providing a reliable foundation for downstream applications.

Deep learning-based point cloud upsampling methods have advanced considerably in recent years [7,8]. Early approaches such as PU-Net [9] and PU-GAN [10] demonstrated the feasibility of generating dense point sets from sparse inputs using feature expansion and adversarial training. Subsequent works improved local geometric modeling via graph convolutions [11], self-attention mechanisms [12], and multi-scale feature aggregation [13]. More recent methods have explored implicit neural representations [14] and diffusion-based generation [15] to recover fine-grained structures. Despite these advances, nearly all existing upsampling methods share a common limitation: they operate globally and uniformly, densifying the entire point cloud without discrimination. A few methods, such as SPU+ [16], introduce semantic awareness by leveraging category-level labels to guide part-aware upsampling. However, they still rely on predefined semantic classes and fully supervised training, lacking the ability to respond to open-vocabulary, user-specified natural language instructions. Moreover, they cannot perform zero-shot localization of novel part concepts or selectively enhance only task-relevant regions on demand.

To emulate this biomimetic principle of language-guided selective perception, we introduce PartSPUNet. Inspired by this biomimetic mechanism of language-guided perceptual resource allocation, this novel framework integrates self-supervised, text-driven 3D semantic segmentation with point cloud upsampling enhanced by a multimodal large language model. The proposed architecture operates through two collaborative modules: a pretrained multimodal semantic segmentation module and a self-supervised multimodal local upsampling module. PartSPUNet processes two primary inputs: a sparse point cloud and a natural language phrase specifying a target point cloud part. Initially, the pretrained cross-modal semantic segmentation module leverages its open-vocabulary understanding, derived from large-scale image–text pretraining, to associate the textual prompt with the corresponding geometric region in the 3D data. It thereby generates a precise semantic mask that isolates the relevant point subset, all without requiring task-specific fine-tuning. Subsequently, this segmented point cluster and its associated textual prompt are fed into a local upsampling module. This module is explicitly designed for geometry-aware densification, jointly interpreting the linguistic semantics and the local geometric structure to synthesize a high-density, detail-rich point cloud within the target region. Crucially, the upsampling operation is confined exclusively to this user-specified component, thereby eliminating redundant computation over the entire point cloud. This selective approach not only elevates the reconstruction quality in the area of interest but also enhances overall computational efficiency. Finally, the enhanced dense local patch is seamlessly reintegrated with the original global point cloud through geometric alignment.

Our contributions are summarized as follows:

- **A Biomimetic Framework for Language-Guided Perception:** We propose PartSPUNet, the first self-supervised point cloud refinement framework that emulates the human cognitive process of language-guided selective attention. It enables operators to intuitively specify regions of interest via natural language for targeted geometric enhancement.
- **An Efficient and Decoupled Architecture:** Our design decouples global segmentation from local upsampling. This allows the high-precision generation module to activate only within user-specified regions, significantly enhancing detail in key areas while improving computational efficiency.
- **Competitive Performance on Local Reconstruction:** Extensive experiments demonstrate that PartSPUNet achieves superior reconstruction fidelity specifically within the user-defined semantic regions, outperforming existing upsampling methods.

## 2. Relate Work

*Deep Learning-Based Point Cloud Upsampling:* Deep learning has brought a major change to how we make point clouds denser. This work started with PU-Net [9], which was the first to use a design called PointNet++ [17]. It learned to add more points by looking at features step by step. A common problem with early methods was that the results looked too smooth and lacked fine details. To fix this, later models like PU-GAN [10] and PUFA-GAN [18] introduced adversarial training, significantly enhancing the realism and visual quality of generated points. Building upon this, the focus shifted toward more sophisticated local geometric modeling. MPU [19] looked at features at different sizes, while PU-GCN [11] explicitly captured neighborhood relations using graph convolutional networks. Concurrently, PU-CRN [20] and PU-Transformer [21] employed self-attention mechanisms to model both local and global dependencies within the point cloud. A pivotal advancement was the integration of semantic awareness into the upsampling process. SPU [7] and SPU+ [16] jointly optimized for density and semantic segmentation, using label priors to guide coherent part-aware generation. In a parallel geometric direction, PUGeo-Net [8] ensured accurate surface reconstruction through a differential parameterization approach. Alongside these developments, the paradigm of implicit neural representations emerged as a powerful alternative. Methods like Neps [14], PU-VoxelNet [22], and PU-Mask [23] learned continuous implicit functions of the underlying surface to generate uniformly dense point sets. For dynamic scenes, frameworks such as SPRGAN [24], VPU [25], and SPU [26] leveraged temporal information by aggregating features across consecutive frames. Most recently, the field has embraced diffusion models for their strong generative capability. Techniques including PDANS [27], SuperPC [28], PUDM [29], and PU-Flow [30] formulate upsampling as a conditional denoising process, iteratively refining noise into detailed structure. This approach excels at recovering high-frequency details, demonstrates robustness to imperfect inputs, and inherently supports multi-scale generation.

*Visual–language contrast pretraining:* Recent works have demonstrated the transferability of vision–language models to three-dimensional understanding. Notably, CLIP [31,32], trained with contrastive learning on extensive image–text pairs, established a robust joint embedding space with strong zero-shot generalization. Inspired by this, researchers have explored adapting its semantic priors to 3D data. CLIP-PCQ [33] investigates the transfer of CLIP’s representations for language-guided 3D understanding. Another approach, adopted by methods like CLIP-GS [34], involves rendering 3D shapes into multi-view images and aligning the rendered features with text via CLIP’s image encoder. A significant limitation of such methods is their dependence on rendering quality, which can obscure the original geometric topology. To establish a direct link between raw point clouds and language, recent frameworks employ point cloud–text contrastive learning. MV-CLIP [35] aligns point clouds with CLIP’s visual branch by projecting them into multi-view depth maps or voxel grids. In a parallel development, Point-BERT [36] and ULIP-2 [37] pretrain dedicated point cloud encoders on large-scale 3D datasets and align their output embeddings with CLIP’s textual space to obtain generalizable joint representations. Despite these advances, existing CLIP-based 3D alignment techniques [6,38,39] have primarily been applied to holistic tasks such as classification, retrieval, or global shape editing. They have not yet been systematically adapted for the precise, local geometric refinement required in tasks like point cloud upsampling. Building upon this foundation, our research takes a significant step forward by leveraging these pretrained point cloud–text alignment models as zero-shot semantic localizers. These models can accurately identify and extract user-specified body parts from sparse point clouds based solely on open-vocabulary textual descriptions, thereby directly enabling targeted upsampling. This approach establishes an end-to-end pathway from free-form language instructions to the precise geometric enhancement of specific regions for the first time.

## 3. Method

**Figure 1:**
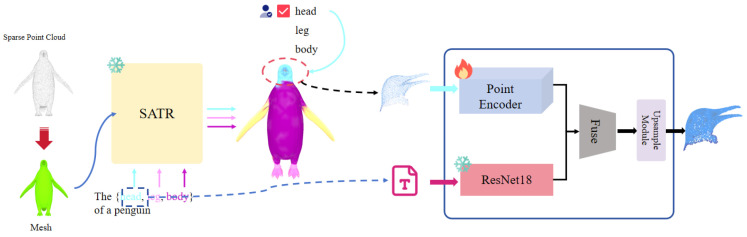
PartSPUNet network framework.

As shown in Figure 1, given a sparse point cloud $P_{s}$ and a natural language query *T* specifying a target body part, our method first applies a zero-shot localization module based on a pretrained vision–language model (CLIP), which was trained on large-scale image–text pairs and requires no fine-tuning on 3D data. This module encodes $P_{s}$ into a geometric feature representation and computes its cross-modal similarity with CLIP-aligned text embeddings of *T*. The process yields a binary semantic mask *M*, which is used to extract the corresponding local point subset $P_{l}\subset P_{s}$.

The extracted subset $P_{l}$ and its associated text prompt *T* are then fed into a self-supervised multimodal upsampling network. This network employs a cross-attention mechanism to fuse linguistic features from a frozen text encoder with the learnable local geometric features of $P_{l}$. Conditioned on this fused representation, the network synthesizes a dense, detail-rich output $P_{dense}$ for the target region. Finally, the enhanced local component $P_{dense}$ is geometrically aligned and merged with the remaining, unmodified portion of the original point cloud, denoted as $P_{u}=\frac{P_{s}}{P_{l}}$. The result is the final, complete upsampled point cloud Po, which preserves the global structure while achieving high-fidelity, language-guided local refinement.

### 3.1. Semantic-Guided Region Localization

To achieve zero-shot localization of linguistically specified regions, we employ a rendering-based strategy that aligns 3D geometry with text in the semantic space of a pretrained CLIP model ([32,36]). The core process involves: (1) generating multi-view 2D representations of the input 3D point cloud; (2) encoding both these views and the text prompt using the frozen CLIP encoders; and (3) aggregating view-specific semantic scores back to 3D points to infer a segmentation mask.

Specifically, given the sparse input point cloud $P_{s}\in R^{N\times 3}$ and a text prompt *T*, we first render *V* view-specific feature maps. Unlike methods that render RGB images, we render depth maps and surface normal maps from *V* uniformly distributed virtual cameras surrounding the point cloud centroid. This choice provides a geometry-centric representation that is robust and aligns well with the structural priors captured by CLIP.

Let the rendered image for view *v* be Iv. A frozen CLIP image encoder $\phi_{img}\left(\cdot \right)$ extracts a global visual feature vector $Z_{v}=\phi_{img}\left(I_{v}\right)\in R^{d_{clip}}$ from each $I_{v}$. Simultaneously, the text prompt *T* is encoded by the frozen CLIP text encoder $\phi_{text}\left(\cdot \right)$ into a normalized semantic embedding: $e_{t}=\frac{\phi_{text}\left(T\right)}{{∥\phi_{text}∥}_{2}}$. The key step is to back-project the 2D semantic information to the original 3D points. For a point $p_{i}\in P_{s}$, we identify the set of views $V_{i}$ in which it is visible. Its semantic affinity score $s_{i}$ is computed as the average cosine similarity between $e_{t}$ and the image features of all views where $p_{i}$ is visible:

(1) $$\begin{array}{cc} s_{i}=\frac{1}{\left|V_{i}\right|}\sum_{v\in V\left(i\right)}z_{v}^{T}e_{t}\; & i=1,\ldots ,N \end{array}$$

$s_{i}$ reflects the consistency between the local geometry around $p_{i}$ (as represented from multiple viewpoints) and the textual concept *T*. A binary segmentation mask $M\in {\left\{0,1\right\}}^{N}$ is then obtained by thresholding these scores:

(2) $$M_{i}=\left\{\begin{array}{cc} 1, & \begin{array}{cc} if & s_{i}\geq \tau \end{array}\\ 0, & otherwise \end{array}\right\}$$

The threshold τ is a hyperparameter. We selected τ based on its performance on a validation set, trading off the completeness of the localized region against the risk of including unrelated points. In practice, we find that a fixed value of τ=0.25 works well across diverse object categories and text prompts, eliminating the need for per-instance tuning. Finally, the target point subset is extracted as:

(3) $$P_{l}=\left\{p_{i}\in P_{s}|M_{i}=1\right\}$$

The computed mask *M* serves a dual purpose in PartSPUNet. Primarily, it directly extracts $P_{l}$ as the region of interest for the subsequent local upsampling module. Furthermore, the continuous scores $s_{i}$ can be utilized as an attention guidance signal within the upsampling network, encouraging the model to focus its capacity on refining areas with high semantic relevance to the text prompt. The entire localization process is zero-shot and training-free. It relies solely on the pretrained cross-modal alignment capabilities of CLIP, requiring no 3D annotations or fine-tuning on point cloud data. This design enables PartSPUNet to respond to open-vocabulary language instructions and provides precise geometric conditioning for the dedicated local upsampling stage.

### 3.2. Text and Point Cloud Feature Fusion

The localized point cloud $P_{l}\in R^{N_{p}\times 3}$ and its corresponding text prompt *T* are fed into a dedicated multimodal upsampling module. The goal is to generate a dense point cloud $P_{part}\in R^{N_{p}\times 3}$r=4, where *r* is the upsampling factor obtained by deeply integrating linguistic semantics with local geometric context. The process consists of three stages: multimodal feature extraction, cross-modal fusion, and geometry-aware feature refinement. We first encode the text prompt and the point cloud into their respective feature spaces.

Textual Feature Extraction: The input prompt *T* is encoded using the same frozen CLIP text encoder $\phi_{text}\left(\cdot \right)$, ensuring feature consistency. To obtain a richer and more adaptable representation, the resulting CLIP text embedding is projected through a lightweight, learnable multi-layer perceptron (MLP) $\psi \left(\cdot \right)$:

(4) $$e=\psi \left(\phi_{text}\left(T\right)\right)\in R^{D}$$

This projection enhances the model’s sensitivity to fine-grained semantic distinctions between prompts targeting different body parts.

Geometric Feature Extraction: The point cloud $P_{l}$ is processed by a multi-scale graph convolutional network. This network employs neighborhood aggregation with varying radii to capture hierarchical geometric information. A larger radius captures broad contextual information for perceiving overall part shape, while a smaller radius precisely models high-frequency details like surface curvature. Feature residual connections and positional encoding are incorporated to stabilize training and preserve spatial information across layers. The output is a multi-scale geometric feature matrix $F_{geo}\in R^{N_{p}\times C}$.

**Figure 2:**
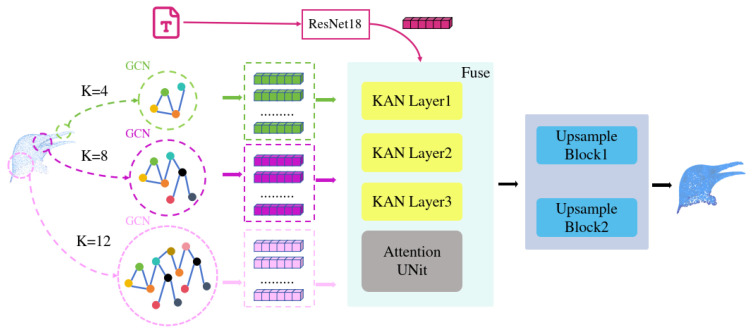
Upsampling network module.

Cross-Modal Fusion via Adaptive Attention: To establish a dynamic, point-wise correspondence between language and geometry, we introduce a fusion module based on the Kolmogorov–Arnold Network (KAN) paradigm (shown in Figure 2). Unlike simple concatenation, this module learns to weight the relevance of the textual semantics for each individual point based on its local geometric context. First, the geometric features $F_{geo}$ are projected to match the textual feature dimension: $F=W_{g}F_{geo}+b_{g}\in R^{N_{p}\times 3}$. A per-point attention weight vector $s_{i}\in R^{1\times D}$ towards the text embedding *e* is then computed via a learnable KAN-inspired function $K\left(\cdot \right)$:

(5) $$s_{i}=\sigma \left(K\left(f_{i},e\right)\right)$$

Here, σ is the sigmoid function, $f_{i}$ is the feature of the $i_{th}$ point, and *K* models a complex, learnable interaction. The final fused feature for each point is generated by modulating the text embedding with this attention weight:

(6) $$f_{fuse,i}=s_{i}⊙e+f_{i}$$

where ⊙ denotes element-wise multiplication. This yields the fused cross-modal feature matrix $F_{fused}\in R^{N_{p}\times 3}$.

### 3.3. Geometry-Aware Feature Refinement

The cross-modal fused feature matrix $F_{fused}\in R^{N_{p}\times 3}$ may contain discontinuities or inconsistencies in regions with low point density, sharp geometric transitions, or ambiguous semantic boundaries. To ensure that the conditioning signal for the subsequent upsampling stage is both smooth and geometrically faithful, we apply a geometry-aware feature refinement module. This module performs spatially adaptive feature aggregation within the local neighborhood of each point, explicitly leveraging the underlying 3D structure to smooth noise while preserving geometric detail.

The refinement process for a point $p_{i}$ with its fused feature vector $f_{i}\equiv F_{fuse}\left[i,:\right]$ is defined as a weighted aggregation over its local neighborhood. The core operation can be decomposed into three key components: neighborhood definition, adaptive weight computation, and normalized aggregation. For a query point $p_{i}\in P_{l}$, we define its local neighborhood $N_{i}$ as the set of its k nearest neighbors within $P_{l}$ based on Euclidean distance. This set provides the local geometric context for feature smoothing. The contribution of a neighbor $p_{j}$ to the refined feature of $p_{i}$ is governed by a data-dependent kernel function $k_{\theta }$. This kernel computes a scalar weight $w_{ij}$ by evaluating the relationship between the two points. It considers not only their spatial displacement $\Delta p_{ij}=p_{i}-p_{j}$ but also their respective feature vectors $f_{i}$ and $f_{j}$:

(7) $$w_{ij}=k_{\theta }\left(△p_{ij},f_{i},f_{j}\right)$$

Here, $k_{\theta }$ is implemented as a small, fully-connected neural network with parameters θ. Its design ensures the weight is sensitive to both geometric proximity and feature-level similarity, allowing it to adaptively smooth features along perceptually coherent regions. The refined feature $f_{i}^{\prime }$ for point $p_{i}$ is computed as the normalized weighted sum of the linearly transformed features of all points in its neighborhood $N_{i}$:

(8) $$f_{i}^{\prime }=\frac{\sum_{j\in N_{j}}w_{ij}\cdot \left(W_{c}f_{j}\right)}{\sum_{j\in N_{i}}w_{ij}+\epsilon }$$

Here, $W_{c}\in R^{D\times D}$ is a learnable linear projection matrix shared across all points. It transforms the fused features before aggregation. The denominator normalizes the weighted sum using the sum of all kernel weights for neighbors of $p_{i}$, ensuring that the aggregation is stable and scale-invariant. A small constant ϵ is added for numerical stability. The output is the refined feature vector $f_{i}^{\prime }$, which captures a smoother, more context-aware representation of the local region centered at $p_{i}$.

Applying this operation to all points $i=1,\ldots ,N_{p}$ in parallel yields the final refined feature matrix $F_{refine}\in R^{N_{p}\times D}$. This matrix serves as the enhanced conditioning signal that guides the coordinate regression in the subsequent point cloud density enhancement module, ensuring that the upsampled points respect both the local geometry and the global semantic intent expressed by the text prompt. 

### 3.4. Loss Function

To train the proposed PartSPUNet in a self-supervised manner, we design a reconstruction objective that enforces geometric fidelity without requiring ground-truth dense point clouds. Given a sparse input point cloud $P_{s}$ and a text prompt *T*, our network generates the final upsampled output $P_{o}$, which comprises the enhanced target region Pdense and the unmodified remainder $P_{u}$. To create a self-supervised training signal, we apply a stochastic downsampling operator $D\left(\cdot ;m\right)$ that randomly selects m points from a given point cloud. The core idea is to encourage the network’s dense output $P_{o}$, when downsampled to the original input size, to reconstruct the spatial distribution of Ps. Formally, the downsampled output is denoted as $D\left(P_{o};\left|P_{s}\right|\right)$. We adopt the Chamfer Distance (CD) as the primary loss function to measure the discrepancy between two point sets. For two point clouds $A=\left\{a_{i}\right\}$ and $B=\left\{b_{j}\right\}$, the CD is defined as:

(9) $$L_{cd}\left(A,B\right)=\frac{1}{\left|A\right|}\sum_{a\in A}\min_{b\in B}{∥a-b∥}_{2}^{2}+\frac{1}{\left|B\right|}\sum_{b\in B}\min_{a\in A}{∥b-a∥}_{2}^{2}$$

This loss is symmetric, differentiable with respect to point coordinates, and permutation-invariant, making it suitable for comparing unordered point sets. The total self-supervised reconstruction loss for our framework is:

(10) $$L_{total}=L_{cd}\left(P_{s},D\left(P_{o};\left|P_{s}\right|\right)\right)$$

Minimizing $L_{total}$ trains the network to produce a dense point cloud $P_{o}$ whose underlying surface is consistent with the sparse observation $P_{s}$.

## 4. Experiment

### 4.1. Experimental Setup

*Parameter settings.* Our model is implemented in PyTorch 2.3.1 and trained using an NVIDIA GeForce RTX 3090 GPU (24GB memory) under the Ubuntu 20.04 operating system. We employ the Adam optimizer with parameters $\beta_{1}=0.9$ and $\beta_{2}=0.999$. The initial learning rate for the main upsampling network is set to $10^{-3}$. The training runs for a total of 51 epochs, with the learning rate decayed exponentially every 20 epochs after the 20th epoch to stabilize convergence.

*Dataset.* We evaluate our method on two standard benchmarks for point cloud upsampling. The PU-GAN dataset contains 147 3D models spanning diverse object categories (e.g., chairs, tables, airplanes), divided into 120 training and 27 testing samples. Each model is processed to generate sparse inputs of 2048 points and corresponding ground-truth dense clouds of 8192 points. The PU1K dataset is more extensive, comprising 1147 models with greater shape and categorical variety, offering a more challenging testbed for point cloud upsampling. In addition, to evaluate the generalization capability of our method to real-world indoor scenes, we also conduct experiments on the Scannet dataset. Scannet contains 1513 dense 3D reconstructions of real indoor environments, covering diverse room layouts and object categories (e.g., chairs, tables, beds, bookshelves). Following the preprocessing methods of prior works, we extract partial point clouds from the original meshes by simulating sensor viewpoints to produce sparse inputs of approximately 2048 points per scene. The corresponding ground-truth dense point clouds are sampled from the original high-resolution meshes at 8192 points.

*Evaluation metrics.* The Chamfer Distance (CD) loss and the Hausdorff Distance (HD) loss are two widely used metrics in point cloud upsampling, reconstruction, and matching tasks, employed to quantify the geometric discrepancies between a source point cloud and a target point cloud. The lower the CD and HD values, the better the upsampling performance. The P2F metric, on the other hand, measures the similarity of distances between pairs of points; similarly, the ↓ lower this metric is, the better the results.

*Comparison Methods.* We compare PartSPUNet against a range of state-of-the-art methods on the PU1K and PU-GAN datasets. The baselines include both supervised approaches (PU-Net, MPU, PU-GAN, Dis-PU, PU-GCN, PU-CRN) and self-supervised approaches (SAPCU, SPU-Net, SPU-PMD, SPU-IMR). For fairness, we use official pretrained models when available. Otherwise, models are retrained from scratch using their publicly released code and recommended settings.

### 4.2. Quantitative and Qualitative Experiment

**Figure 3:**
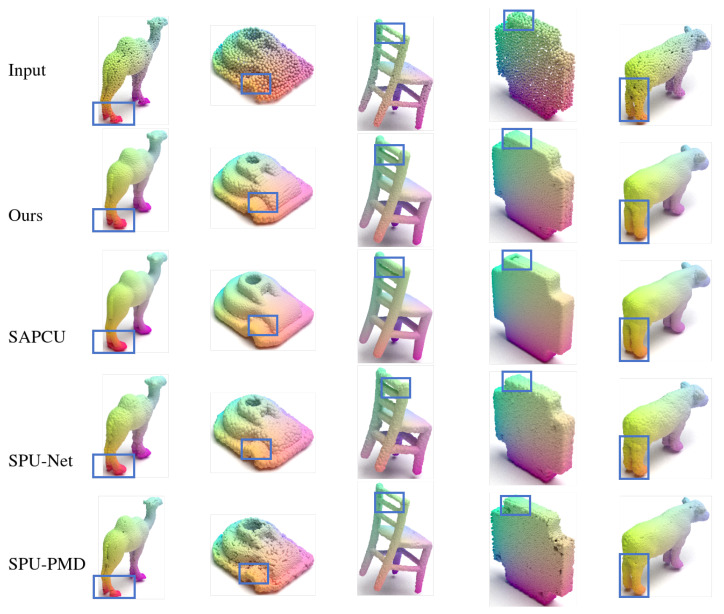
A visual comparison of the proposed method with existing self-supervised point cloud upsampling methods on PUGAN.

*PUGAN Dataset.* Figure 3 and Table 1 show quantitative and qualitative results for several representative categories from the PU-GAN dataset. When compared with existing methods, our approach demonstrates reasonable performance in generating point clouds with acceptable geometric quality and local structural coherence. Some competitive methods may encounter challenges in certain cases, such as partial recovery of slender structures (e.g., camel legs), minor discontinuities or slight deformations around delicate components (e.g., thin chair slats), and non-uniform point distributions near sharp boundaries. Our method shows improvements in maintaining local continuity and geometric integrity, providing smoother transitions, and achieving relatively uniform point distributions across surfaces.

**Figure 4:**
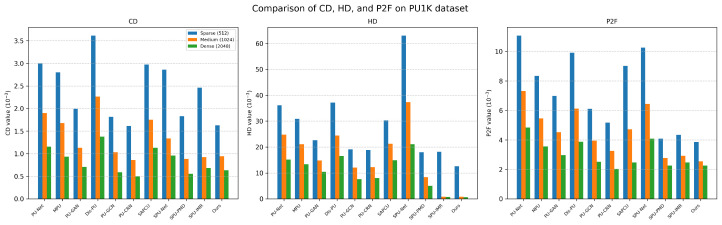
Bar chart comparison of the three metrics (CD, HD, and P2F) of different methods on the PU1K dataset.

**Figure 5:**
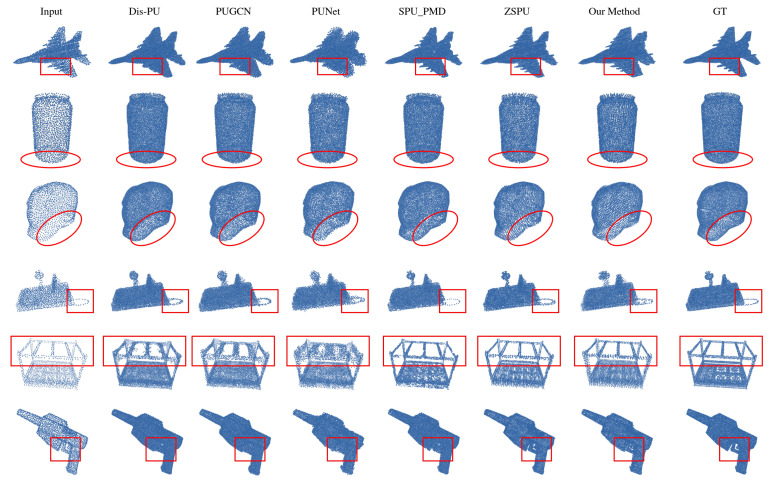
Visualizing and comparing the performance of existing supervised and self-supervised point cloud upsampling methods on the PU1K dataset.

*PU1K dataset.* We further evaluated our model on the PU1K dataset (shown in Table 2), which features greater shape diversity and includes a wide range of object categories such as airplanes, bottles, head models, boats, tables, firearms, and more. As shown in Figure 4 and Figure 5, our method consistently delivers strong performance across all categories and shows remarkable robustness. Compared to existing baselines, the point clouds generated by our approach have sharper edges and cleaner structural boundaries, effectively reducing common artifacts like edge blurring, surface fragmentation, and topological inconsistencies. On curved surfaces such as bottle bodies or human heads, our model produces smooth natural curvatures without introducing high-frequency noise.

**Figure 6:**
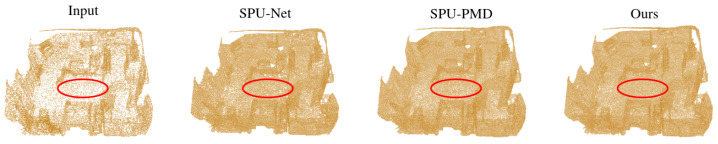
Visualizing and comparing the performance of existing self-supervised point cloud upsampling methods on the scannet dataset.

*Scannet Dataset.* Figure 6 compares upsampling results on a real-world indoor scan from Scannet. The sparse input lacks sufficient detail on chair backs, table edges, and wall boundaries. SPU-Net produces dense but over-smoothed outputs with noticeable outliers. SPU-PMD yields more structured results yet suffers from surface discontinuities and fragmentation. In contrast, PartSPUNet generates uniformly dense points on planar regions, preserves sharp edges and thin structures, and maintains geometric continuity even in occluded areas, demonstrating robust generalization to authentic robotic perception scenarios.

### 4.3. Intuitive Text Interaction Methods for Robotic Perception

**Figure 7:**
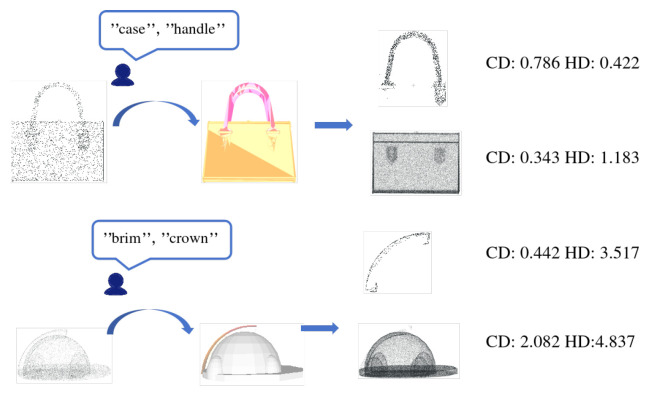
Text-guided point cloud upsampling effect on the object point cloud.

**Figure 8:**
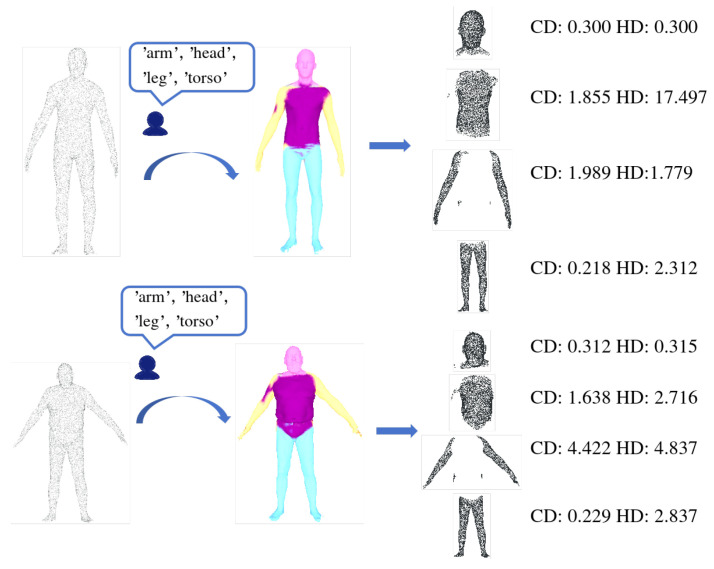
Text-guided point cloud upsampling results on a human body point cloud.

To thoroughly evaluate the proposed text-driven local upsampling framework, we conducted visualization experiments on two representative categories: human bodies and generic objects. As shown in Figure 7, the raw input for a human scan provides only a coarse torso outline with nearly no points in the facial region; nevertheless, leveraging a zero-shot semantic segmentation module—built upon multi-view rendering and a frozen CLIP encoder—our system accurately localizes the “head” and generates a precise binary mask without any training on human data. Similarly, for a handbag (Figure 8), where the input “handle” consists of just a few nearly collinear points lacking visible curvature, our model reconstructs a continuous, naturally curved handle with smooth axial transitions that faithfully match its real-world geometry, demonstrating strong open-vocabulary adaptability and structure-awareness. Although these evaluations are performed on standard 3D reconstruction benchmarks (PU1K and PU-GAN), they directly reflect real-world robotic perception challenges: the sparse inputs simulate the low-resolution, partial observations commonly caused by sensor noise, occlusion, or limited field-of-view in practical robot deployments. Critically, PartSPUNet’s ability to faithfully reconstruct user-specified semantic parts—such as “hands,” “wheels,” or “connectors”—addresses a core need in robotics: recovering high-fidelity geometry exactly where it matters for task execution. For instance, in a human–robot collaboration scenario, an operator could verbally instruct the robot to “pay attention to the damaged area,” and our method would enable on-demand, localized reconstruction without reprocessing the entire scene—representing a significant step toward interactive, semantics-aware robotic perception systems grounded in biomimetic principles.

### 4.4. Robust Experiment Analysis

**Figure 9:**
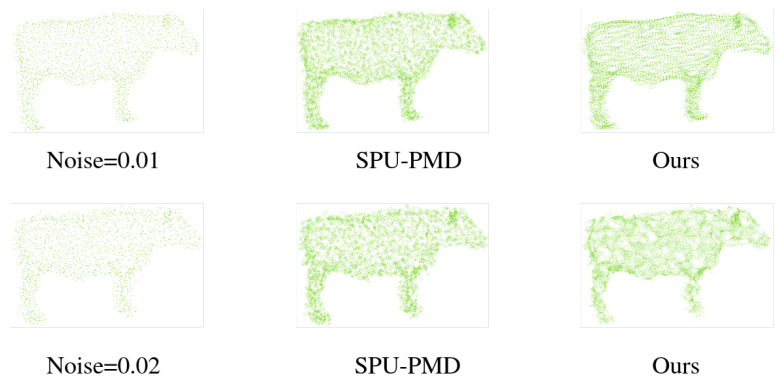
This visualization experiment compares the performance of our method and SPU-PMD in text-guided local point cloud upsampling under low-noise (noise=0.01) and high-noise (noise=0.02) inputs.

*Noise Test:* Under low noise (noise=0.01, top row), our method faithfully reconstructs fine structures (shown in Figure 9). Under high noise (noise=0.02, bottom row), while global shape and semantic consistency (guided by “cow head”) are preserved, localized artifacts appear: over-smoothing in sparse regions, spurious clusters near edges, and minor topological distortions. In contrast, the SPU-PMD baseline, lacking explicit semantic guidance, fails catastrophically under high noise, producing geometrically plausible but semantically incorrect structures (e.g., generating points outside the target “head” region). Quantitatively, our method significantly outperforms SPU-PMD under noise=0.02. This demonstrates that our text-guided localization provides crucial semantic robustness, preventing misplacement even when geometric fidelity degrades.

**Figure 10:**
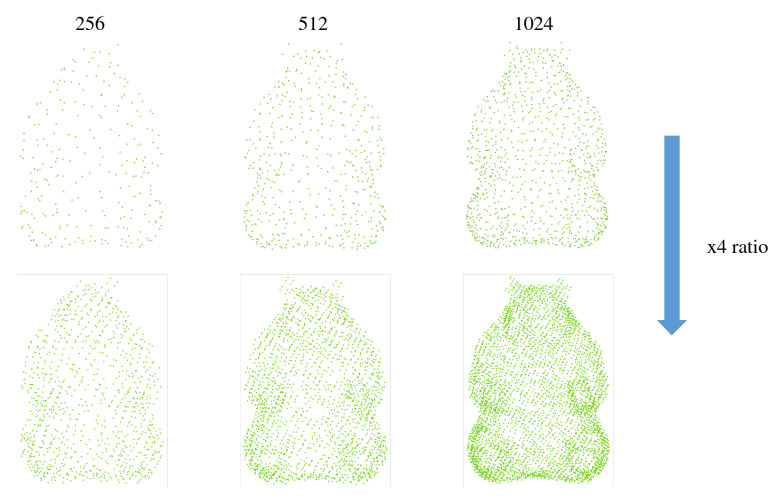
The qualitative results of our upsampling method across varying input densities.

*Different Point Cloud Input:* Figure 10 presents a qualitative comparison of the upsampling performance under varying input densities, where the top row visualizes the sparse source point clouds and the bottom row displays the corresponding 4 × upsampled results generated by our framework. The visualization demonstrates the model’s robustness in recovering high-fidelity geometry from extremely sparse inputs, as the reconstructed point clouds in the lower row consistently exhibit a dense, uniform distribution that accurately preserves the underlying object topology and fine-grained structural details observed in the sparse references above.

### 4.5. Ablation Experiment

To validate the effectiveness of the key components in the proposed method, we conducted a systematic ablation study on PartSPUNet. All experiments were performed under the same dataset and training settings.

*Components of the upsampling module:* We first analyzed the two core components of PartSPUNet. As shown in Table 3 when KAN is removed (w/o KAN), CD, HD, and P2F rise to 0.451, 2.864, and 2.849, respectively, showing that KAN plays a critical role in modeling complex nonlinear mappings between geometry and semantics. In contrast, removing only the attention mechanism causes a smaller performance drop, yet the resulting model still falls significantly short of the full version. This indicates that the attention mechanism effectively strengthens alignment between local geometric details and textual semantics.

*Multimodal Alignment Strategies:* To further investigate the impact of feature fusion strategies, we conduct an ablation study on different approaches for integrating text and point cloud features (results are shown in Table 4). A variant that completely discards textual input (“w/o Text”) leads to a noticeable performance drop, highlighting the importance of language guidance in shape generation. We subsequently examine several fixed fusion ratios between text and point cloud features, denoted as Text: Point (α:β), where α and β represent the weights assigned to each modality. The results indicate that performance fluctuates with different fixed ratios, yet none surpass the proposed adaptive fusion mechanism.

## 5. Discussion of Failure Cases

While our PartSPUNet framework demonstrates promising performance in text-guided local point cloud upsampling, we acknowledge several limitations and failure cases that warrant discussion. First, the semantic localization module, which relies on a zero-shot vision–language model (CLIP), occasionally fails to accurately identify the target region when presented with ambiguous or overly generic textual prompts (e.g., “handle” in complex mechanical assemblies with multiple handles). In such cases, the subsequent upsampling is applied to incorrect regions, leading to geometric artifacts. Second, our upsampling module struggles with extremely sparse input regions (fewer than 10 points), where the lack of structural information hinders the generation of plausible high-resolution geometry. Third, the method exhibits sensitivity to severe noise or outliers in the input point cloud, which can propagate through the upsampling process and degrade output quality. Finally, when the text prompt contains domain-specific terminology not well-represented in CLIP’s training corpus, the localization accuracy drops significantly. These limitations highlight important directions for future work: (1) developing more robust prompt disambiguation mechanisms, (2) incorporating explicit geometric priors to handle extremely sparse inputs, (3) integrating denoising modules as a preprocessing step, and (4) exploring domain-adaptive fine-tuning of the vision–language backbone.

## 6. Conclusions

In this work, we presented PartSPUNet, a novel framework for text-guided local refinement of sparse point clouds. By leveraging pretrained vision–language models, our method enables intuitive, semantic-driven control over the geometric densification process without requiring paired training data. More importantly, PartSPUNet embodies a biomimetic design principle: it operationalizes the biological concept of language-guided selective perception, allowing a robotic system to dynamically allocate its perceptual resources to behaviorally relevant regions based on high-level language instructions. Through extensive experiments, including a dedicated evaluation on the PU1K and PUGAN datasets, we demonstrated that our approach not only achieves state-of-the-art reconstruction quality but also directly enhances the performance of core robotic perception pipelines. This capability is crucial for next-generation biomimetic robots that must interact with complex, unstructured environments with human-like adaptability and precision. Future work will focus on deploying PartSPUNet on physical robotic platforms for real-time closed-loop manipulation tasks and exploring its integration with active perception strategies to further emulate the efficiency of biological sensing systems.

## Figures and Tables

**Table 1:** Comparison of CD, HD, and P2F metrics on the PUGAN dataset with existing point cloud upsampling methods (unit: $10^{-3}$). √ means is a the supervised method, × means is a self-supervised method.

Method	Ground Truth	CD ↓	HD ↓	P2F ↓	Params (KB) ↓
PU-Net	√	0.817	11.150	7.838	814.0
MPU	√	0.713	10.614	5.381	76.2
PU-GAN	√	0.469	8.220	4.047	684.2
PU-GCN	√	0.401	5.630	3.650	76.0
PU-CRN	√	0.289	4.175	2.369	969.9
SAPCU	×	0.443	10.397	3.446	8718.3
SPU-Net	×	0.509	23.497	6.106	778.5
SPU-PMD	×	0.328	3.420	2.226	1166.5
SPU-IMR	×	0.361	3.762	2.449	44,473.2
Ours	×	0.380	2.264	2.364	567

**Table 2:** Comparison of CD, HD, and P2F metrics on the PU1K dataset with existing point cloud upsampling methods (unit: $10^{-3}$). √ means is a the supervised method, × means is a self-supervised method.

Method	Ground Truth	Sparse (512) Input			Medium (1024) Input			Dense (2048) Input		
		CD ↓	HD ↓	P2F ↓	CD ↓	HD ↓	P2F ↓	CD ↓	HD ↓	P2F ↓
PU-Net	√	2.999	36.129	11.077	1.899	24.754	7.321	1.155	15.170	4.847
MPU	√	2.803	30.843	8.334	1.679	21.119	5.450	0.935	13.327	3.560
PU-GAN	√	1.991	22.642	6.979	1.132	14.809	4.530	0.707	10.411	2.963
Dis-PU	√	3.616	37.134	9.911	2.265	24.455	6.120	1.380	16.524	3.880
PU-GCN	√	1.817	19.153	6.104	1.035	12.032	3.946	0.585	7.577	2.504
PU-CRN	√	1.611	18.835	5.161	0.861	12.214	3.246	0.499	8.068	2.027
SAPCU	×	2.973	30.237	9.030	1.754	21.292	4.712	1.130	14.903	2.462
SPU-Net	×	2.863	63.031	10.262	1.338	37.368	6.444	0.955	21.058	4.083
SPU-PMD	×	1.831	17.955	4.082	0.881	8.321	2.765	0.554	5.030	2.254
SPU-IMR	×	2.461	18.213	4.335	0.926	8.442	2.914	0.683	7.090	2.469
Ours	×	1.628	12.594	3.852	0.942	8.372	2.547	0.632	6.041	2.247

**Table 3:** Experiment results of different modules on PUGAN dataset (unit: $10^{-3}$).

Methods	CD ↓	HD ↓	P2F ↓
w/o KAN	0.451	2.864	2.849
w/o Attention	0.408	2.640	2.644
Ours (Full)	0.380	2.264	2.364

**Table 4:** Experiment results of different alignment ratios on PUGAN dataset (unit: $10^{-3}$).

Methods	CD ↓	HD ↓	P2F ↓
w/o Text	0.425	2.512	2.603
Text: Point (2:8)	0.412	2.437	2.521
Text: Point (4:6)	0.395	2.355	2.442
Text: Point (6:4)	0.399	2.381	2.493
Text: Point (8:2)	0.405	2.390	2.484
Ours (Full)	0.380	2.264	2.364

## Data Availability

The data presented in this study are available on request from the corresponding author due to the relevant data and code are still being organized and will be published on GitHub in the future.
